# Effects of microalgae supplementation on performance, egg quality, yolk fatty acid levels, and blood parameters in laying hens: a meta-analysis

**DOI:** 10.1038/s41598-025-28515-3

**Published:** 2025-12-29

**Authors:** Moh Sofi’ul Anam, Uswatun Muslykhah, Muhammad Maulana Sadid, Cuk Tri Noviandi, Andriyani Astuti, Dimas Hand Vidya Paradhipta, Ali Agus

**Affiliations:** 1 Department of Animal Nutrition and Feed Science, Faculty of Animal Science, Universitas Gadjah Mada, Yogyakarta, 55281 Indonesia; 2 Tropical Feed Resources Research and Development Center (TROFREC), Department of Animal Science, Faculty of Agriculture, Khon Kaen University, Khon Kaen, 40002 Thailand

**Keywords:** Egg quality, Laying hens, Microalgae, N-3 polyunsaturated fatty acids, Performance, Biochemistry, Physiology, Plant sciences

## Abstract

Microalgae have gained attention in laying hen nutrition due to their richness in n-3 polyunsaturated fatty acids (PUFA), pigments, and antioxidants. However, their efficacy in poultry diets remains inconsistent. This meta-analysis quantitatively evaluated the effects of dietary microalgae supplementation on performance, egg quality, yolk fatty acid composition, and blood biochemistry in laying hens. Thirty-six peer-reviewed articles were retrieved from Scopus, ScienceDirect, PubMed, and Google Scholar following PRISMA guidelines, and effect sizes were calculated using weighted mean differences. Microalgae supplementation did not affect feed intake (*p* > 0.05) but significantly improved hen-day egg production, egg weight, egg mass, yolk weight, shell traits, and Haugh units (*p* < 0.05). Yolk pigmentation increased in color intensity and redness (*p* < 0.001) but decreased in lightness (*p* < 0.001). Microalgae enhanced yolk total n-3 PUFA, eicosapentaenoic acid, and docosahexaenoic acid (*p* < 0.001), while reducing n-6 PUFA and the n-6/n-3 ratio (*p* < 0.05). Blood total cholesterol, aspartate aminotransferase, and alanine aminotransferase concentrations decreased (*p* < 0.05). In conclusion, dietary microalgae supplementation augments laying performance, enhances egg quality and yolk pigmentation, fortifies yolk with beneficial n-3 fatty acids, and promotes liver health, highlighting its potential as a functional feed additive in layer diets.

## Introduction

Eggs from laying hens are widely regarded as a high-quality food source, offering a well-balanced profile of proteins, lipids, vitamins, and minerals, all of which are vital for human nutrition^1^. Consistent with global population growth, the demand for table eggs has steadily risen over the recent decades^2^. This increased demand places added pressure on poultry production systems to not only maximize egg output but also enhance egg nutritional quality, aligning with contemporary consumer preferences for functional and health-promoting foods^1,3^.

Conventional poultry diets typically lack sufficient levels of bioactive compounds such as n-3 polyunsaturated fatty acids (PUFAs), essential amino acids, pigments, and antioxidants, all of which are crucial for optimizing animal performance and improving the health-promoting qualities of animal-derived products. For instance, the fatty acid profile of conventional eggs is often considered suboptimal due to low concentrations of long-chain n-3 PUFAs, particularly docosahexaenoic acid (DHA, C22:6 n-3) and eicosapentaenoic acid (EPA, C20:5 n-3), which are known for their cardiovascular and cognitive health benefits in humans^4,5^. Moreover, continued dependence on conventional feed ingredients has raised both economic and ecological concerns, including fluctuating feed costs, increased land use pressure, and the potential for environmental degradation^6,7^. These challenges highlight the urgent need for nutrient-dense and sustainable feed alternatives that can improve both the productivity and resilience of laying hens^8^.

Among the array of promising alternatives, microalgae have emerged as highly functional feed ingredients. Microalgae are unicellular, photosynthetic microorganisms that inhabit a wide range of aquatic environments, including both marine and freshwater ecosystems. Notable species include *Arthrospira platensis*, *Chlorella vulgaris*, and *Schizochytrium* sp., which are each characterized by a distinct nutrient composition^9^. These microalgae are recognized for their rich content of long-chain n-3 fatty acids, carotenoids, chlorophylls, vitamins, minerals, and potent antioxidants, positioning them as ideal candidates for enhancing the nutritional value of poultry diets^10–12^. Additionally, microalgae cultivation offers an environmentally sustainable and resource-efficient approach, requiring minimal arable land and freshwater inputs, and is capable of utilizing industrial by-products as substrates, thereby supporting the advancement of a circular bioeconomy^13,14^.

In laying hens, microalgae supplementation has demonstrated several physiological benefits. These alterations include improved intestinal morphology, enhanced nutrient absorption, favorable gut microbiota modulation, and elevated systemic antioxidant capacity^15,16^. Bioactive components such as phycocyanin, carotenoids, and n-3 fatty acids have been shown to facilitate immune function and oxidative balance while also promoting increased egg production, yolk pigmentation, Haugh unit, and yolk DHA/EPA enrichment^17–19^. Further studies have reported improvements in blood lipid profiles, antioxidant enzyme activities, and hormonal parameters related to reproduction and metabolism^20,21^.

Despite these encouraging results, the findings remain inconsistent across studies. While certain trials reported significant improvements in feed efficiency and egg quality traits, others observed minimal or even adverse effects on performance indicators, such as feed intake, feed conversion ratio (FCR), and laying rate^22–24^. Contradictions have also been noted regarding yolk fatty acid composition, antioxidant biomarkers, and immune response outcomes^16,25,26^. The observed heterogeneity may be attributed to variations in microalgal species, inclusion levels, processing methods, supplementation duration, hen strain, and basal diet composition^27^.

To resolve these inconsistencies and develop a more integrated understanding of microalgae’s effects on layer production, a comprehensive meta-analysis was conducted. Meta-analysis is a robust statistical tool that aggregates findings across independent studies to refine effect size estimation, identify sources of variability, and support evidence-based decisions^28–30^. Although several narrative reviews have explored the role of microalgae in poultry systems^10,12,31,32^, there remains a lack of meta-analytic studies that systematically evaluate their effects specifically in laying hens. Accordingly, this study aimed to address this knowledge gap by performing a quantitative evaluation of the impact of microalgae supplementation on laying hen performance, egg quality traits, yolk fatty acid profile, and key blood biochemical indicators.

## Materials and methods

### Literature search and eligibility criteria

**Fig. 1:**
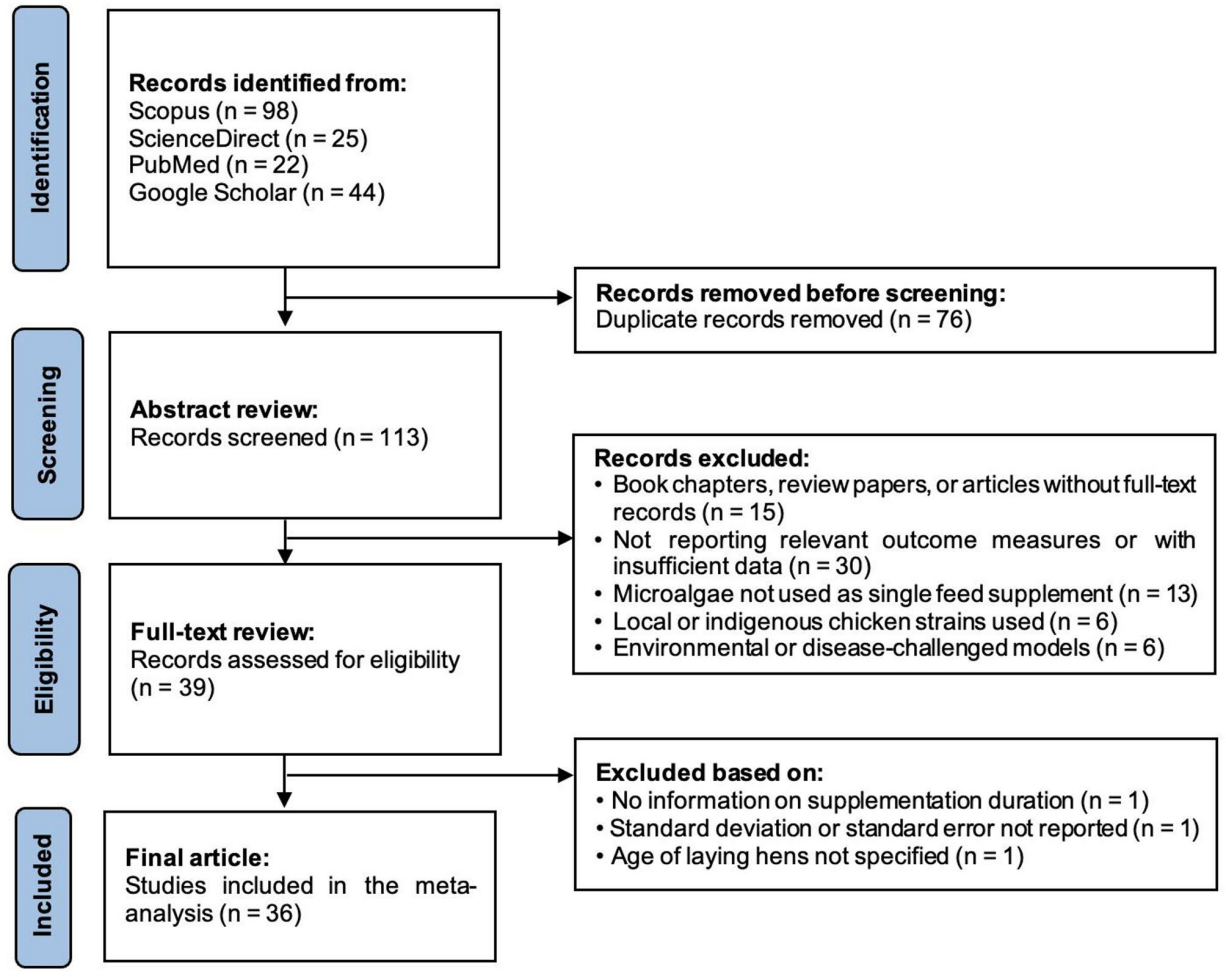
Flow diagram illustrating the article selection process.

The research question was formulated employing the PICO strategy outlined by Nishikawa-Pacher^33^, in which P represents the population, I the intervention, C the comparison, and O the outcome. In the current study, the study population was comprised laying hens; the intervention included dietary supplementation with microalgae; the comparison was between microalgae-supplemented and non-supplemented diets; and the outcomes assessed included treatment means for performance, egg quality, yolk fatty acids, and blood biochemistry. Subsequently, the scientific documents that examined the effects of microalgae dietary supplementation in laying hens were identified, selected, and included in the database in accordance with PRISMA guidelines^34^, as depicted in Fig. 1. The literature was identified through systematic searches conducted across the search engines Scopus, ScienceDirect, PubMed, and Google Scholar, with no restriction on publication year. The search strategy combined keywords associated with laying hens and microalgae species frequently utilized in poultry nutrition (e.g., “microalgae,” “laying hen,” “egg quality,” “*Chlorella*,” “*Arthrospira*,” “*Spirulina*,” “*Nannochloropsis*,” “*Schizochytrium*,” “*Phaeodactylum*,” “*Isochrysis*,” “*Aurantiochytrium*”)^31^.

Systematic searches yielded 189 scientific documents. After excluding the duplicates, 113 documents remained for screening. Studies were excluded if they exhibited any of the following characteristics: (1) conference proceedings, books, theses, review articles, or non-peer-reviewed materials; (2) studies involving non-commercial laying hens (native/backyard/village chickens were explicitly excluded); and (3) studies that utilized microalgal extracts or oils instead of whole microalgae powder or combined microalgae with other functional additives. The remaining documents were subjected to rigorous evaluation, and only those meeting all of the following inclusion criteria were retained for the final analysis: (1) full-text original research articles published in English and peer-reviewed; (2) studies involving healthy, commercial laying hens under standard conditions in randomized experiments; (3) studies that compared the effect of dietary supplementation with whole microalgae powder against a control treatment (diets lacking microalgae supplementation), with both groups receiving the same basal diet; (4) studies reporting data on parameters related to laying hen performance, egg quality, yolk fatty acids, and/or blood biochemistry; (5) studies that clearly specified the microalgae species used, supplementation dose, and duration of the experimental period; and (6) studies that provided essential statistical parameters, including standard deviation (SD), standard error of the mean (SEM), number of replicates (n), and means for both microalgae-supplemented and control groups.

### Data extraction

After excluding duplicates and an initial screening of titles and abstracts, 39 full-text articles were selected for comprehensive evaluation. Among these, 36 studies fulfilled all predefined inclusion criteria and were subsequently incorporated into the meta-analysis dataset (Table 1). Data from each eligible publication were meticulously extracted and organized using the Microsoft Excel software. Extracted information included study location (country, continent), laying hen characteristics (strain, age, sample size), duration of the feeding trial, type of basal diet, microalgae species, inclusion level, and dietary nutrient composition (metabolizable energy, crude protein, ether extract, crude fiber, lysine, and methionine).

**Table 1:** Details of the studies reviewed in the meta-analysis. N = number of comparisons; total hen = number of hens in the treatment group; DoS = days of supplementation; SBM = soybean meal; ^1^provided as raw, untreated biomass; ^2^provided as fermented product; ^3^provided as defatted product; ^4^provided as full-fat product: ^5^provided as by-product; ^6^provided as commercial DHA-gold product.

No	Studies	Country	Continent	*N*	Total hen	Strain	Age, week	DoS	Type of diet	Species	Levels (%)
1	Fredriksson et al.^116^	Sweden	Europe	5	15	Hy-Line W98	55	28	Wheat + Barley + Oat	*Nannochloropsis oculata* ^1^	0–20
2	Halle et al.^62^	Germany	Europe	26	104	Lohmann Brown	22	240	Corn + SBM	*Chlorella vulgaris* ^1^	0–0.75.75
3	Zahroojian et al.^117^	Iran	Asia	4	128	Hy-Line W36	63	25	Wheat + SBM	*Arthrospira plantensis* ^1^	0–2.5.5
4	Jeon et al.^15^	Korea	Asia	5	200	Hy-Line Brown	70	42	Corn + Wheat + SBM	*Chlorella vulgaris* ^1^	0–0.5.5
5	Zheng et al.^16^	China	Asia	4	108	Hy-Line Brown Brown	40	42	Corn + SBM	*Chlorella vulgaris* ^2^	0-0-2
6	Bruneel et al.^22^	Belgium	Europe	4	12	ISA Brown	25	28	Corn + Wheat + SBM	*Nannochloropsis gaditan* ^1^	0–10
7	Englmaierová et al.^52^	Czech Republic	Europe	6	120	ISA Brown	25–39	84	Corn + Wheat + SBM	*Chlorella* sp.^1^	0–1.25.25
8	Kotrbáček et al.^118^	Czech Republic	Europe	8	32	Hisex Brown	56	54	Corn + Wheat + SBM	*Chorella* sp. ^1^	0–2
9	Lemahieu et al.^54^	Belgium	Europe	8	72	ISA Brown	29	28	Not reported	*Phaeodactylum tricornutum*^1^, *Nannochloropsis oculate*^1^, *Isochrysis galbana*^1^, *Chlorella fusca*^1^	0–0.25.25
10	Zahroojian et al.^24^	Iran	Asia	4	128	Hy-Line W36	63	28	Wheat + SBM	*Arthrospira plantensis* ^1^	0–0.25.25
11	An et al.^119^	Korea	Asia	5	100	Hy-Line brown	70	14	Corn + Wheat + SBM	*Chorella vulgaris* ^1^	0–1
12	Lemahieu et al.^120^	Belgium	Europe	8	72	ISA Brown	33	21	Corn + Wheat + SBM	*Isochrysis galbana* ^1^	0–8.1.1
13	Ao et al.^121^	USA	America	6	120	Hy-Line W36	36	32	Corn + SBM	*Schizochytrium limacinum* ^1^	0–3
14	Ekmay et al.^106^	USA	America	5	90	Shaver White	26	14	Corn + Wheat + SBM	*Desmodesmus* sp.^3^, *Staurosira* sp.^4^	0–0.025.025
15	Kim & Kang^122^	Korea	Asia	4	160	Hy-Line Brown	28	56	Corn + SBM	*Chorella* spp.^5^	0–7.5.5
16	Park et al.^18^	Korea	Asia	12	216	ISA Brown	40	42	Corn + Wheat + SBM	*Schizochytrium* sp. ^1^	0–1
17	Lemahieu et al.^123^	Belgium	Europe	12	36	ISA Brown	27	21	Corn + Wheat	*Isochrysis galbana*^1^, *Nannochloropsis oculata* ^1^	0–4
18	Neijat et al.^124^	Canada	America	10	40	Lohman LSL-Classic	34	42	Corn + Wheat + SBM	*Schizochytrium* sp.^6^	0–3.36.36
19	Ahammed et al.^125^	Bangladesh	Asia	6	96	Shaver-579 Brown	78	49	Corn + SBM	*Arthrospira plantensis* ^1^	0–0.3.3
20	Manor et al.^64^	USA	America	10	50	Shaver White	46	42	Corn + SBM	*Nannochloropsis oceanica* ^1^	0–23
21	Omri et al.^21^	Tunisia	Africa	15	45	Lohman White	44	42	Corn + SBM	*Arthrospira plantensis* ^1^	0–2.5.5
22	Wu et al.^19^	China	Asia	6	360	Lohmann Brown	37	28	Corn + SBM	*Nannochloropsis* sp.^1^	0–8
23	Yonke & Cherian^58^	USA	America	7	28	White Leghorn	24	112	Corn + SBM	*Schizochytrium* sp.^6^	0–1
24	Liu et al.^20^	China	Asia	8	360	Hy-Line Brown	26	70	Corn + SBM	*Aurantiochytrium* sp.^1^	0–1
25	Curabay et al.^126^	Turkey	Asia	5	60	Lohman LSL-Classic	60	28	Wheat + SBM	*Arthrospira plantensis* ^1^	0–2
26	Rey et al.^26^	Spain	Europe	3	54	White Leghorn, Rhode Island Red	23	21	Corn + SBM	*Arthrospira plantensis* ^1^	0–3
27	Tufarelli et al.^53^	Iran	Asia	8	216	Hy-Line W36	63	84	Corn + SBM + Wheat	*Arthrospira plantensis* ^1^	0–2
28	Abbas et al.^17^	Saudi Arabia	Asia	5	250	Hy-Line W36	40	70	Corn + SBM + Wheat	*Arthrospira plantensis* ^1^	0–12
29	Mens et al.^25^	Netherlands	Europe	6	192	Super Nick	25	28	Corn + Wheat + SBM + Sunflower Meal	*Nannochloropsis limnetica* ^1^	0–3
30	Kim et al.^23^	Korea	Asia	3	54	Hy-Line Brown	21	28	Corn + SBM	*Chlorella vulgaris* ^1^	0–0.5.5
31	Panaite et al.^127^	Romania	Europe	20	120	Lohman Brown	38	42	Corn + Wheat	*Chlorella vulgaris*^1^, *Arthrospira plantensis* ^1^	0–2
32	Poveda-Víquez et al.^68^	Costa Rica	America	5	80	ISA Brown	52	28	Corn + SBM	*Arthrospira maxima* ^1^	0–6
33	Wahyuni et al.^128^	Indonesia	Asia	6	72	ISA Brown	81	35	Corn + Rice Bran + SBM	*Arthrospira plantensis* ^1^	0–0.3.3
34	Kiran et al.^129^	Pakistan	Asia	3	150	Hy-Line Brown	30	52	Corn + SBM	*Schizochytrium limacinum* ^1^	0–1
35	Madacussengua et al.^130^	Spain	Europe	12	48	Brown Nick	19	112	Corn + SBM	*Chlorella vulgaris* ^1^	0–10
36	Salahuddin et al.^60^	USA	America	8	192	White Leghorn	39	42	Corn + SBM	*Arthrospira platensis* ^1^	0–1

Outcome variables were classified into four major domains: (1) performance parameters, including feed intake, HDEP, FCR, egg weight, and egg mass; (2) egg quality traits, such as yolk weight, albumen weight, shell weight, yolk ratio, albumen ratio, shell ratio, shell thickness, shell strength, Haugh unit, yolk index, and yolk cholesterol; (3) yolk color attributes, including subjective yolk color intensity (determined using a color scale) and objective color measurements (L* [lightness], a* [redness], b* [yellowness]); (4) yolk fatty acids composition, including n-3 and n-6 PUFAs, n-6/n-3 ratio, ALA, C18:3 n-3), EPA, and DHA; and (5) blood biochemical parameters, including total protein, triglycerides, total cholesterol, aspartate aminotransferase (AST), and alanine aminotransferase (ALT).

For each treatment group, the arithmetic means and associated variability metrics (SD or SEM) were calculated. When only SEM data were reported, SD was estimated using the formula: $\:\mathrm{S}\mathrm{D}=\mathrm{S}\mathrm{E}\mathrm{M}\:\times\:\:\sqrt{\mathrm{n}}$ ​, where n represents the number of replicates or experimental units. Only response variables reported in a minimum of three different scientific articles were included in the meta-analysis^35,36^. Summary details of the study characteristics are summarized in Table 1, while the descriptive nutrient profiles of the diets are illustrated in Table 2.

**Table 2:** Overview of the nutritional composition of the experimental diets used in the meta-analysis. N = number of samples; Max = maximum; Min = minimum; SD = standard deviation.

Nutrient composition of diets	*N*	Min	Max	Mean	SD
Metabolizable energy, kcal/kg	95	2581.20	3011.40	2785.37	83.36
Crude protein, %	110	14.60	19.60	16.61	1.14
Ether extract, %	54	3.00	6.80	4.49	1.07
Crude fiber, %	46	1.88	7.00	3.04	1.16
Lysine, %	65	0.69	1.05	0.85	0.10
Methionine, %	34	0.34	0.51	0.41	0.06

### Statistical analysis, heterogeneity, and publication bias

All statistical analyses were conducted employing the “meta”^37^ and “metafor”^38^ packages in R software (version 4.5.0). The effects of dietary microalgae supplementation were measured using weighted mean differences (WMD), comparing treatment and control groups. The WMD was selected as the effect size metric due to its clear interpretability and robust statistical validity^39^. To account for inter-study variations, a random-effects model employing inverse-variance weighting was applied^40^. Heterogeneity among studies was investigated using Cochran’s Q-test and the *I²* statistic, with *I²* values above 50% and Q-test *p*-values below 0.05 indicating substantial heterogeneity^35,41^. To ascertain potential publication bias, Egger’s regression test^42^ and Begg’s rank correlation test^43^ were conducted, with *p* < 0.05 considered statistically significant. Additionally, sensitivity analysis was performed using a leave-one-out meta-analysis to evaluate the influence of individual studies on the overall estimate^44^.

### Meta-regression and subgroup analyses

Univariate meta-regression analysis was conducted to examine whether differences in response variables could be explained by specific study-level moderators, including strain of laying hens, duration of microalgae supplementation, inclusion level, and microalgae species. Meta-regression analyses were limited to outcome variables that fulfilled the following criteria: (1) reported in atleast ten independent studies; (2) exhibited significant heterogeneity (Q-test *p* < 0.05, *I²* > 50%)^35,41^; and (3) demonstrated no indication of publication bias based on Egger’s and Begg’s tests (*p* > 0.05)^42,43^. Moderator variables were grouped as follows: (1) laying hen strain (e.g., Lohman Brown, ISA Brown, Lohman Classic); (2) supplementation duration (≤ 50 days vs. >50 days); (3) algae inclusion level (< 1%, 1%–5%, > 5% of feed); and (4) microalgae species (e.g., *Nannochloropsis limnetica*, *Aurantiochytrium* sp., *C. vulgaris*). When a moderator demonstrated a statistically significant effect (*p* < 0.05), subgroup analyses were performed to further examine differences in effect sizes across categories. To ensure analytical robustness, only subgroups with at least three comparisons were included in the analysis^45^.

## Results

### Characteristics of the included studies

This meta-analysis encompassed 36 peer-reviewed studies conducted across 20 countries spanning four continents: Asia (58.3%), Europe (27.8%), America (11.1%), and Africa (2.8%) (Table 1). Collectively, the database comprised 5,358 laying hens, representing a diverse range of commercial strains. Hy-Line Brown and Hy-Line W36 were the most commonly utilized strains (52.8%), followed by ISA Brown, Lohmann Brown, and White Leghorn. The experimental period ranged from 14 to 240 days, with supplementation initiated in birds aged between 19 and 81 weeks. Basal diets were predominantly formulated using corn, wheat, and soybean meal, reflecting standard commercial feeding practices.

All included studies administered microalgae in dehydrated meal or powder form, with dietary inclusion levels ranging from 0.025% to 12%. However, most studies reported inclusion levels between 0.1% and 3%. The types of microalgae used encompassed a broad taxonomic range, including *Nannochloropsis oculata*,* C. vulgaris*,* S. plantensis*,* Nannochloropsis gaditan*,* Chlorella* sp., *Phaeodactylum tricornutum*,* Nannochloropsis oculate*,* Isochrysis galbana*,* Chlorella fusca*, *Schizochytrium limacinum*,* Desmodesmus* sp., *Staurosira* sp., *Schizochytrium* sp., *Nannochloropsis oceanica*,* Nannochloropsis* sp., *Aurantiochytrium* sp., *N. limnetica*,* Arthrospira maxima*, and *Schizochytrium limacinum*. A total of 398 treatment comparisons were extracted and classified into five major outcome domains: performance, egg quality, yolk fatty acid profile, yolk color, and blood parameters.

Additional descriptive statistics detailing the nutrient profiles reported in the included studies are presented in Table 2. On average, the diets provided 2,785.37 kcal/kg of metabolizable energy, with crude protein levels ranging from 14.60% to 19.60% (mean: 16.61%). The levels of ether extract, crude fiber, lysine, and methionine concentrations were within the recommended ranges for laying hens and conformed to the NRC^46^ nutrient guidelines.

### Performance

As illustrated in Table 3, dietary microalgae inclusion exerted no significant impact on feed intake or FCR (*p* > 0.05). Conversely, hen-day egg production (HDEP) exhibited a significant improvement in response to microalgae supplementation (*p* < 0.001).

**Table 3:** Effects of dietary microalgae supplementation on laying Hen performance parameters.

Item	Unit	NS	NC	Microalgae		Heterogenity		Bias (*p*-value)	
				WMD (95% CI)	*p*-value	*I* ^*2*^	*p*-value^1^	ET	BT
Feed intake	g	28	77	0.071 (−0.889, 1.093)	0.840	99.66	< 0.001	0.025	0.961
HDEP	%	28	76	1.826 (1.042, 2.61)	< 0.001	99.99	< 0.001	0.194	0.348
FCR	-	20	53	−0.022 (−0.053, 0.01)	0.173	99.29	< 0.001	0.013	0.678

NS = number of studies; NC = number of comparisons between microalgae and control treatments; WMD = weighted mean differences between treatments with microalgae and control; CI = confidence interval of WMD; ^1^ = *p*-value to cochran’s Q statistics; *I*^*2*^< = proportion of total variation of size effect estimates that is due to heterogeneity; ET = Egger’s regression asymmetry test; BT = Begg’s rank correlation method; HDEP = Hen-day egg production; FCR = feed conversion ratio.

### Egg quality traits

Table 4 indicates that microalgae-supplemented diets significantly improved several egg quality parameters, including egg weight, egg mass, yolk weight, albumen weight, shell strength, and Haugh units (*p* < 0.05). Furthermore, significant increases were observed in both shell weight and shell thickness (*p* < 0.01). Regarding yolk pigmentation, both yolk color intensity and redness increased significantly, while lightness decreased (*p* < 0.001). In contrast, no statistically significant alterations were detected in yolk cholesterol, yolk ratio, albumen ratio, shell ratio, or yolk yellowness (*p* > 0.05).

**Table 4:** Impact of dietary microalgae supplementation on egg quality traits in laying hens.

Item	Unit	NS	NC	Microalgae		Heterogenity		Bias (*p*-value)	
				WMD (95% CI)	*p*-value	*I* ^*2*^	*p*-value^1^	ET	BT
Egg weight	g	34	96	0.247 (0.004, 0.489)	0.046	99.93	< 0.001	0.013	0.927
Egg mass	g	15	33	1.084 (0.144, 2.023)	0.024	99.08	< 0.001	0.703	0.686
Yolk weight	g	15	45	0.260 (0.026, 0.493)	0.029	98.24	< 0.001	0.716	0.084
Albumen weight	g	9	26	0.701 (0.051, 1.351)	0.035	97.95	< 0.001	0.019	0.859
Shell weight	g	9	25	0.152 (0.038, 0.266)	0.009	96.65	< 0.001	0.055	0.381
Yolk ratio	%	9	29	0.298 (−0.052, 0.647)	0.095	97.38	< 0.001	0.865	0.619
Albumen ratio	%	7	18	−0.107 (−0.564, 0.350)	0.646	96.46	< 0.001	0.116	0.588
Shell ratio	%	9	23	0.240 (−0.026, 0.505)	0.077	98.63	< 0.001	0.001	0.285
Shell thickness	mm	20	52	0.010 (0.003, 0.017)	0.003	99.85	< 0.001	0.231	< 0.001
Shell strenght	kg/cm^2^	12	27	0.118 (0.008, 0.228)	0.036	99.12	< 0.001	0.617	0.412
Haugh unit	-	20	47	1.191 (0.284, 2.098)	0.010	98.98	< 0.001	0.496	0.010
Yolk cholesterol	mg/g	7	19	0.404 (−0.231, 1.039)	0.212	97.33	< 0.001	0.664	0.671
Yolk color intensity	-	28	79	2.548 (1.862, 3.233)	< 0.001	99.99	< 0.001	0.220	0.000
Yolk lightness	-	13	42	−4.626 (−6.12, −3.132	< 0.001	99.82	< 0.001	0.001	0.075
Yolk redness	-	13	42	6.115 (4.635, 7.595)	< 0.001	99.98	< 0.001	< 0.001	0.820
Yolk yellowness	-	13	42	0.421 (−1.282, 2.125)	0.628	99.89	< 0.001	0.158	0.356

NS = number of studies; NC = number of comparisons between microalgae and control treatment; WMD = weighted mean differences between treatments with microalgae and control; CI = confidence interval of WMD; ^1^ = *p*-value to Cochran’s Q statistics; *I*^*2*^= proportion of total variation of size effect estimates that is due to heterogeneity; ET = Egger’s regression asymmetry test; BT = Begg’s rank correlation method; yolk color intensity = determined using a color scale; lightness = L* value, redness = a*, yellowness = b*

### Yolk fatty acid composition

Dietary microalgae supplementation significantly lowered yolk n-6 PUFA levels (*p* < 0.05) and the n-6/n-3 PUFA ratio (*p* <0.001), while markedly increasing total n-3 PUFA, EPA, and DHA (*p* < 0.001) concentrations. Yolk a-linolenic acid (ALA) levels were not significantly affected by microalgae supplementation (*p* >0.05) (Table 5).

**Table 5:** Effects of dietary microalgae supplementation on yolk fatty acid composition in laying hens.

Item	Unit	NS	NC	Microalgae		Heterogenity		Bias (*p*-value)	
				WMD (95% CI)	*p*-value	*I* ^*2*^	*p*-value^1^	ET	BT
n-6 PUFA	% FA	12	32	−0.883 (−1.592, −0.174)	0.015	99.51	< 0.001	0.021	0.228
n-3 PUFA	% FA	12	33	1.206 (0.751, 1.661)	< 0.001	99.98	< 0.001	< 0.001	0.202
n-6/n-3 PUFA ratio	% FA	11	29	−5.655 (−7.250, −4.061)	< 0.001	99.97	< 0.001	0.990	0.462
ALA	% FA	10	24	0.008 (−0.058, 0.074)	0.819	99.88	< 0.001	0.017	0.708
EPA	% FA	12	30	0.123 (0.062, 0.184)	< 0.001	99.92	< 0.001	< 0.001	0.002
DHA	% FA	13	33	1.669 (1.171, 2.167)	< 0.001	99.99	< 0.001	0.260	0.675

NS = number of studies; NC = number of comparisons between microalgae and control treatment; WMD = weighted mean differences between treatments with microalgae and control; CI = confidence interval of WMD; ^1^ = *p*-value to Cochran’s Q statistics; *I*^*2*^ = proportion of total variation of size effect estimates that is due to heterogeneity; ET = Egger’s regression asymmetry test; BT = Begg’s rank correlation method; PUFA = polyunsaturated fatty acids; FA = fatty acids; ALA = a-linolenic acid (C18:3 n-3); EPA = eicosapentaenoic acid (C20:5 n-3); DHA = docosahexaenoic acid (C22:6 n-3)

### Blood biochemical parameters

As shown in Table 6, dietary microalgae supplementation significantly reduced total cholesterol, AST (*p <* 0.05), and ALT concentrations (*p <* 0.01). No significant effects were observed on total protein or triglyceride levels (*p >* 0.05).

**Table 6:** Effects of dietary microalgae supplementation on the blood profiles in laying hens.

Item	Unit	NS	NC	Microalgae		Heterogenity		Bias (*p*-value)	
				WMD (95% CI)	*p*-value	*I* ^*2*^	*p*-value^1^	ET	BT
Total protein	g/dL	4	12	0.639 (−0.049, 1.326)	0.069	98.60	< 0.001	0.017	0.830
Triglycerides	mg/dL	3	7	9.653 (−6.646, 25.952)	0.246	99.46	< 0.001	0.728	0.748
Total cholesterol	mg/dL	4	9	−7.074 (−13.444, −0.704)	0.030	92.91	< 0.001	0.539	1.000
AST	U/L	4	11	−13.772 (−24.235, −3.310)	0.010	99.93	< 0.001	0.069	0.254
ALT	U/L	4	11	−4.491 (−7.67, −1.311)	0.006	99.86	< 0.001	0.525	0.871

NS = number of studies; NC = number of comparisons between microalgae and control treatment; WMD = weighted mean differences between treatments with microalgae and control; CI = confidence interval of WMD; ^1^ = *p*-value to Cochran’s Q statistics; *I*^*2*^ = proportion of total variation of size effect estimates that is due to heterogeneity; ET = Egger’s regression asymmetry test; BT = Begg’s rank correlation method; AST = aspartate aminotransferase; ALT = alanine aminotransferase

### Heterogeneity, publication bias, and meta-regression

A detailed summary of the heterogeneity metrics and publication bias assessments is presented in Tables 3, 4, 5 and 6. All assessed outcomes demonstrated considerable heterogeneity, with *I²* values exceeding 90% and highly significant Cochran’s Q statistics (*p <* 0.001). Additionally, Egger’s and Begg’s tests indicated the presence of potential publication bias in several response variables (*p <* 0.05). These variables include feed intake, FCR, shell thickness, shell strength, Haugh unit, yolk color intensity, lightness, redness, n-3 PUFA, and EPA. Conversely, no evidence of publication bias (*p >* 0.05 in both tests) was detected in HDEP, egg weight, yolk weight, egg mass, shell weight, albumen weight, yolk ratio, albumen ratio, shell ratio, yolk cholesterol, yellowness, n-6 PUFA, n-6/n-3 PUFA ratio, ALA, DHA, total cholesterol, AST, and ALT. The leave-one-out sensitivity analysis further confirmed the robustness of the pooled WMD estimates, indicating that no individual study substantially influenced the overall effect. Accordingly, meta-regression analyses were conducted exclusively on response variables reported in at least ten independent studies and exhibited no strong indication of publication bias (*p >* 0.05), consistent with the recommendations of Koricheva et al.^47^.

As shown in Table 7, no significant associations (*p >* 0.05) were identified between the duration of microalgae supplementation and any of the analyzed response variables. Strain was identified as a significant moderator for HDEP (*p* = 0.001, R² = 23.59%) and egg mass (*p* = 0.026, R² = 23.45%). Yolk weight variability across studies was significantly influenced by all three moderators—strain (*p <* 0.001, R² = 32.78%), microalgae level (*p* = 0.001, R² = 23.67%), and microalgae species (*p* = 0.017, R² = 22.23%). Shell strength heterogeneity was predominantly affected by strain (*p <* 0.001, R² = 51.53%), microalgae level (*p* = 0.001, R² = 36.38%), and species (*p* = 0.002, R² = 37.55%). For yolk yellowness, both microalgae level (*p* = 0.032, R² = 10.79%) and species (*p* = 0.001, R² = 31.87%) were identified as significant moderators. Regarding yolk fatty acid composition, the n-6/n-3 PUFA ratio was significantly moderated by strain (*p* = 0.004, R² = 33.55%) and microalgae species (*p <* 0.001, R² = 48.85%). Similarly, DHA concentrations were significantly moderated by strain (*p <* 0.001, R² = 41.14%) and microalgae species (*p <* 0.001, R² = 40.95%).

**Table 7:** Meta-regression analysis of the relationship between covariates and measured outcomes.

Outcomes	Covariates	QM	Df	*p*-value	R^2^ (%)
Hen-day egg production	Strain	30.429	10	0.001	23.59
	DoS	3.279	1	0.070	3.45
	Microalgae level	1.786	2	0.409	0.00
	Microalgae species	15.707	14	0.332	2.30
Egg mass	Strain	17.381	8	0.026	23.45
	DoS	0.712	1	0.399	0.00
	Microalgae level	2.13	2	0.345	0.31
	Microalgae species	9.07	6	0.170	8.92
Yolk weight	Strain	30.431	9	< 0.001	32.78
	DoS	0.004	1	0.947	0.00
	Microalgae level	14.825	2	0.001	23.67
	Microalgae species	23.074	11	0.017	22.23
Shell strenght	Strain	30.449	5	< 0.001	51.53
	DoS	2.481	1	0.115	6.02
	Microalgae level	14.914	2	0.001	36.38
	Microalgae species	18.804	5	0.002	37.55
Yolk yellowness (b*)	Strain	34.834	9	< 0.001	39.39
	DoS	2.973	1	0.085	4.73
	Microalgae level	6.915	2	0.032	10.79
	Microalgae species	28.975	10	0.001	31.87
Yolk n-6/n-3 PUFA ratio	Strain	21.057	7	0.004	33.55
	DoS	0.06	1	0.806	0.00
	Microalgae level	2.972	2	0.226	3.39
	Microalgae species	34.521	8	< 0.001	48.85
Docosahexaenoic acid (DHA, C22:6 n-3)	Strain	29.041	7	< 0.001	41.14
	DoS	0.85	1	0.357	0.00
	Microalgae level	0.365	2	0.833	0.00
	Microalgae species	31.119	9	< 0.001	40.95

DoS = duration of supplementation; QM = coefficient of moderators, which is considered significant at *p < *0.05; Df = degree of freedom; R^2^ = the amount of heterogeneity accounted

### Subgroup analysis

**Fig. 2:**
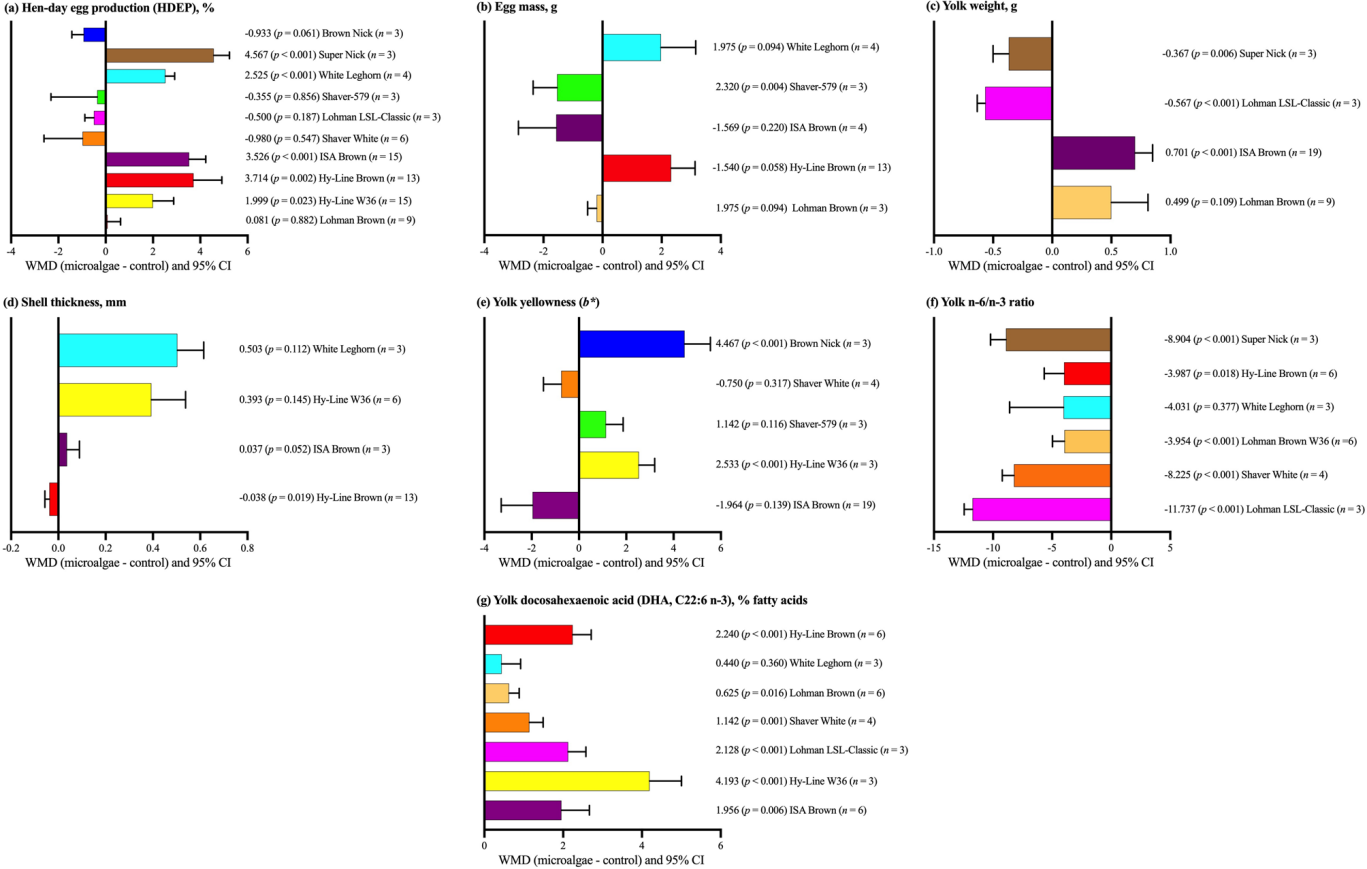
Subgroup analysis (subgroup = strain) of the impact of dietary microalgae supplementation in laying hen diets; WMD = weighted mean differences between microalgae treatment and control groups. Positive WMD values indicate an increase and negative values indicate a decrease in the parameter compared with the control; improvement depends on whether higher or lower values are desirable for each parameter.

As illustrated in Fig. 2a, HDEP was significantly improved in several strains, including Super Nick (*p <* 0.001), White Leghorn (*p* = 0.001), ISA Brown (*p* = 0.001), and Hy-Line Brown (*p* = 0.003) strains. Conversely, other strains such as Brown Nick, Shaver White, Lohman LSL-Classic, Shaver-579, and Lohman Brown demonstrated no statistically significant changes (*p >* 0.05). As depicted in Fig. 2b, a notable increase in egg mass was observed in the Shaver-579 strain (*p* = 0.004). Meanwhile, the remaining strains—White Leghorn, Hy-Line Brown, and Lohman Brown—did not reveal significant alterations (*p >* 0.05). Figure 2c illustrates that yolk weight significantly increased in the ISA Brown strain (*p <* 0.001), whereas significant decreases were observed in Super Nick (*p* = 0.006) and Lohman LSL-Classic (*p <* 0.001) strains. A significant decrease in shell thickness was observed only in Hy-Line Brown (*p* = 0.019), as depicted in Fig. 2d. Hy-Line W36 (*p* = 0.004) and Brown Nick (*p* < 0.001) showed a good response to dietary microalgae in terms of yolk yellowness (Fig. 2e). Significant improvements in yolk n-6/n-3 PUFA ratio were evident in Super Nick, Hy-Line Brown, Shaver White, and Lohman LSL-Classic (*p <* 0.05) (Fig. 2f). Figure 2g highlights a pronounced increase in DHA concentration across several strains, including Hy-Line Brown (*p <* 0.001), Shaver White (*p* = 0.004), ISA Brown (*p* = 0.006), Hy-Line W36 (*p <* 0.001), Lohman Brown (*p* = 0.016), and Lohman LSL-Classic (*p <* 0.001); however, the White Leghorn remained unaffected (*p >* 0.05).

**Fig. 3:**
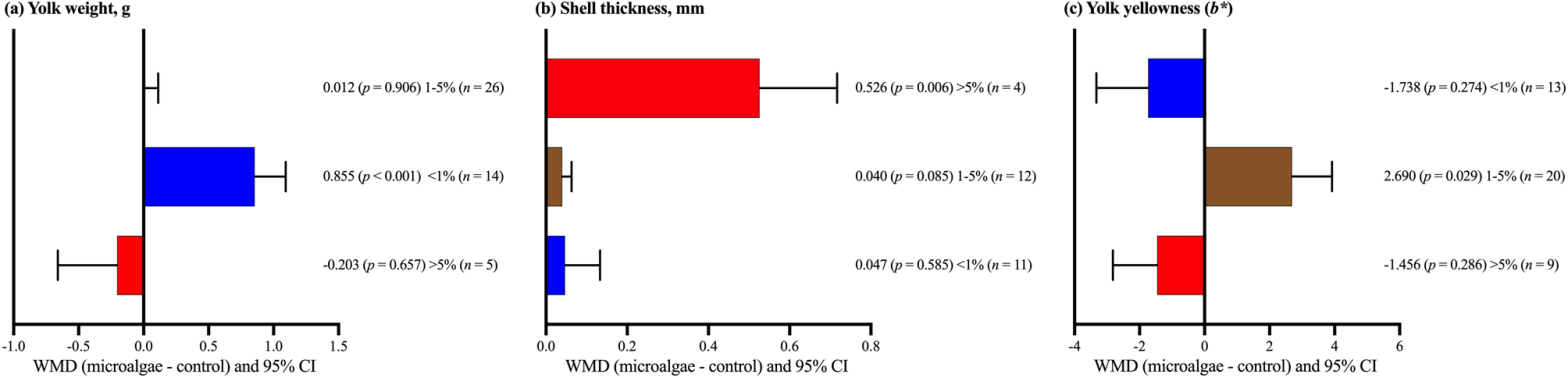
Subgroup analysis (subgroup = microalgae level) of the effect of dietary microalgae supplementation in laying hen diets; WMD = weighted mean differences between microalgae treatment and control groups. Positive WMD values indicate an increase and negative values indicate a decrease in the parameter compared with the control; improvement depends on whether higher or lower values are desirable for each parameter.

Subgroup analysis based on microalgae inclusion level (Fig. 3a) showed a significant increase in yolk weight only at inclusion levels below 1% (*p* < 0.001), while no significant effects were observed at levels 1%–5% and above 5% (*p* > 0.05). In Fig. 3b, the shell thickness was significantly elevated in the presence of > 5% inclusion (*p* = 0.006). As depicted in Fig. 3c, the enhancement of yolk yellowness was significant at the 1%–5% microalgae inclusion level (*p* = 0.029).

**Fig. 4:**
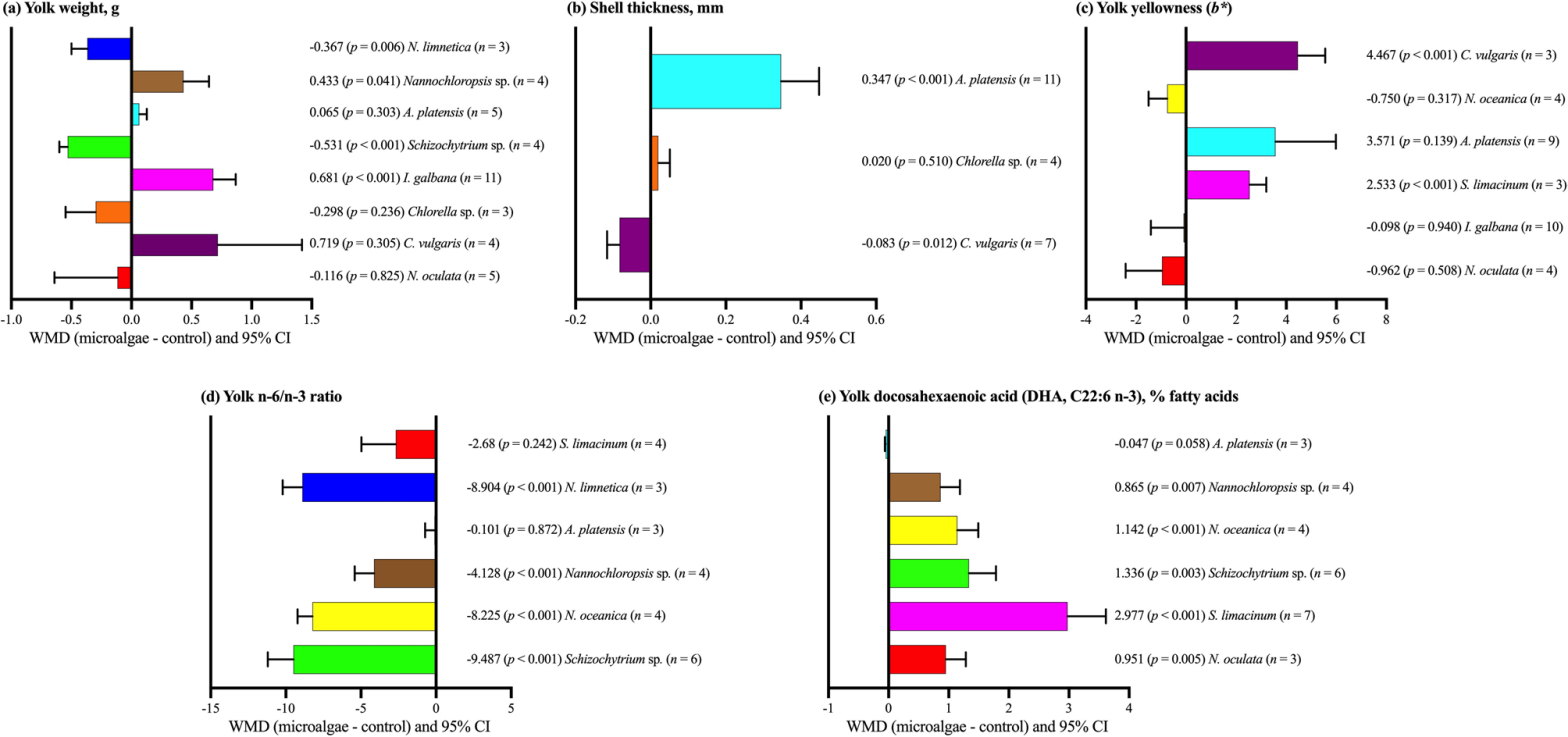
Subgroup analysis (subgroup = microalgae species) of the effect of dietary microalgae supplementation in laying hen diets; WMD = weighted mean differences between microalgae treatment and control groups. Positive WMD values indicate an increase and negative values indicate a decrease in the parameter compared with the control; improvement depends on whether higher or lower values are desirable for each parameter.

A breakdown by microalgae species, shown in Fig. 4, further highlights species-specific responses. Regarding yolk weight (Fig. 4a), significant increases were observed for *I. galbana* (*p <* 0.001) and *Nannochloropsis* sp. (*p* = 0.041), whereas *Schizochytrium* sp. (*p <* 0.001) and *N. limnetica* (*p* = 0.006) demonstrated reductions. Shell thickness (Fig. 4b) was positively affected by *A. platensis* (*p <* 0.001). Significantly improved yolk yellowness was observed in *C. vulgaris* (*p <* 0.001) and *S. limacinum* (*p <* 0.001), both demonstrating strong effects on color intensity (Fig. 4c). The yolk n-6/n-3 PUFA ratio (Fig. 4d) was significantly lowered in *N. limnetica*, *N. oceanica*, *Schizochytrium* sp., and *Nannochloropsis* sp. (*p <* 0.001), while *S. limacinum* and *A. platensis* exhibited no significant effect (*p >* 0.05). Finally, the DHA content in yolk (Fig. 4e) was significantly elevated in *S. limacinum*, *Schizochytrium* sp., *N. oceanica*, *N. oculata*, and *Nannochloropsis* sp. (*p <* 0.01). A marginally significant trend was observed for *A. platensis* (*p* = 0.058).

## Discussion

The current meta-analysis demonstrated that dietary supplementation with microalgae resulted in a significant enhancement in HDEP, whereas no consistent effects were observed for feed intake and FCR. These findings suggest that microalgae boost reproductive efficiency without increasing feed consumption, an outcome that supports cost-effective and sustainable egg production^12^. The neutrality of feed intake responses reveals that the inclusion of microalgae does not disrupt the physiological regulation of voluntary feed intake in laying hens. Birds are known to modify their intake based on metabolic cues rather than dietary novelty, and the presence of bioactive compounds in microalgae does not appear to impede this homeostatic control^48–50^. Multiple studies^17,19,51^, have reported stable feed intake across a range of microalgae species and inclusion levels. This suggests that microalgae, even when abundant in lipid and pigment fractions, do not regulate satiety signaling pathways or induce aversive feeding responses^52,53^.

Furthermore, the inclusion of microalgae supports consistent nutrient assimilation without necessitating increased feed intake, likely due to its high nutrient density and lipid-rich composition, which appear adequate to meet physiological demands even in the presence of rigid cell walls^19,54,55^. Additionally, the inclusion of microalgae enables the dietary provision of highly digestible PUFAs, vitamins, and trace elements that may meet or exceed metabolic demands, further mitigating the need for increased feed consumption^10,23^. The response of FCR to microalgae supplementation was generally neutral across the dataset. This outcome aligns with the observation that, while egg production improved, feed intake remained unchanged. From a physiological standpoint, it is plausible that microalgae augment nutrient partitioning efficiency, particularly in pathways linked to reproduction. However, the FCR metric may not effectively capture such shifts in resource allocation when the overall energy intake and egg mass outputs remain within typical commercial ranges^56,57^.

The most prominent effect of microalgae supplementation was on egg production, as reflected by the significant increase in HDEP. This outcome is likely attributable to the abundance of bioactive compounds present in microalgae, especially n-3 PUFAs such as DHA and EPA. These fatty acids constitute critical components of phospholipid membranes in ovarian and hepatic tissues and also function as precursors for prostaglandins and resolvins that regulate follicular development and ovulation^19,21,58^. In particular, the modulation of arachidonic acid metabolism through dietary n-3 PUFA may promote the transition of the local hormonal milieu toward anti-inflammatory and pro-ovulatory eicosanoid profiles, thus facilitating the follicular recruitment process^26^.

Antioxidant pigments such as lutein, *β*-carotene, and zeaxanthin—all rich in microalgae like *C. vulgaris*, *A. platensis*, and *Schizochytrium* sp.—further promote improved reproductive performance by mitigating ovarian oxidative stress^32,59^. Oxidative damage is linked to impaired follicle viability and decreased reproductive output, whereas dietary antioxidants help counteract lipid peroxidation and preserve granulosa cell integrity^15,60^. Studies by Ismail et al.^61^ and Fernandes et al.^51^ demonstrated elevated ovarian follicle counts and estradiol levels in microalgae-fed hens, along with upregulated expression of antioxidant enzymes such as superoxide dismutase and glutathione peroxidase, furthr reinforcing this mechanistic link. Beyond lipid and antioxidant pathways, micronutrients like vitamin B12 and selenium—naturally present in certain algal species—are essential for reproductive hormone synthesis and immune-endocrine crosstalk. Vitamin B12 acts as a cofactor in methylation pathways involved in steroidogenesis, whereas selenium contributes to thyroid hormone metabolism and redox regulation, both of which are closely related to egg formation and laying persistence^18,62^.

Subgroup analysis revealed that strain significantly moderated the response of HDEP to microalgae inclusion, with White Leghorn and Super Nick laying hens exhibiting greater improvements than Lohman Brown. This differential responsiveness may be attributed to strain-specific variations in ovarian physiology, metabolic rate, and lipid transporter expression, which affect the efficient assimilation and use of bioactive nutrients for egg production^63–65^. These observations align with previous research on functional nutrient supplementation, supporting the concept that genetic background interacts with dietary interventions to regulate performance outcomes^66,67^.

Dietary microalgae supplementation significantly enhanced several egg quality parameters, including egg weight, yolk weight, albumen weight, shell thickness, shell strength, and Haugh unit, while also augmenting yolk pigmentation, as demonstrated by elevated yolk color intensity and redness accompanied by a reduction in yolk lightness. These effects indicate the bioactive complexity of microalgae and their capacity to modulate the physiological pathways regulating egg formation and quality^12^.

The enhancement in egg and yolk weights can be attributed to the distinct lipid and amino acid profiles of microalgae species such as *Schizochytrium* sp., *C. vulgaris*, and *N. oculata*. These microalgae offer highly digestible long-chain PUFAs, notably DHA and EPA, which play dual roles as structural constituents of yolk lipoproteins and as metabolic energy sources that facilitate follicular development and oocyte maturation^19,21^. Supplementation with DHA-rich microalgae promotes a dose-dependent enrichment of yolk DHA content, which may boost yolk mass and improve its nutritional quality^20^. Furthermore, microalgae can preserve or even increase egg weight, even when used to partially replace conventional protein sources like soybean meal, possibly due to their complementary amino acid composition^68^. Species like *Chlorella* and *Arthrospira* are rich in essential amino acids, including arginine, lysine, and methionine, which are crucial for embryonic development, yolk protein synthesis, and reproductive efficiency^69^. Specifically, DHA-enriched yolks exhibit intensified vitellogenesis—a liver-driven process in which dietary lipids are incorporated into very-low-density lipoproteins (VLDL) and vitellogenin. These lipoproteins are generated under the influence of estrogen and are then released into the circulatory system, where they are absorbed by developing ovarian follicles through receptor-mediated endocytosis, promoting yolk formation^70–72^.

Enhancements in albumen weight and Haugh units—two key indicators of internal egg quality—are likely attributable to the antioxidative properties and high-quality protein content of dietary microalgae. Bioactive compounds, including *β*-carotene, tocopherols, and phycocyanin, abundantly present in microalgae, effectively scavenge reactive oxygen species (ROS), thereby lowering the oxidative denaturation of albumen proteins during their synthesis and secretion along the oviductal magnum. This protection is vital for preserving albumin viscosity and minimizing premature protein degradation^60,73,74^. Furthermore, phycocyanobilin and glutathione-like molecules present in microalgae serve as potent superoxide dismutase mimetics that reinforce the redox homeostasis essential for optimal protein folding and gel matrix formation during albumen deposition. These antioxidant compounds stabilize the albumen structure as well as help preserve its height and functional integrity—factors that directly contribute to a higher Haugh unit^75–78^. In addition to their antioxidant content, microalgae supply highly bioavailable proteins and essential amino acids, particularly lysine and methionine, which are vital substrates for albumen protein synthesis. A sufficient supply of these amino acids enables the structural cohesion and viscosity of the albumen matrix, promoting improved internal quality parameters^17,69^.

Shell quality, as revealed by augmented thickness and breaking strength, is improved by the mineral-dense profile of microalgae, which are rich in bioavailable calcium, phosphorus, and magnesium—minerals critical for eggshell calcification^79,80^. These minerals promote the activation of calcium-binding matrix proteins and the enzyme carbonic anhydrase within the shell-forming region, thereby supporting effective calcium carbonate accumulation during eggshell formation^81^. In addition, algal-derived sterols and vitamin D analogs may elevate intestinal calcium absorption and upregulate uterine calcium transporters, facilitating more efficient mineral mobilization during shell formation^82–85^.

The enhancement of yolk pigmentation, particularly in color intensity and redness, is primarily linked to the accumulation of lipid-soluble pigments—including lutein, zeaxanthin, canthaxanthin, and *β*-carotene—derived from microalgae^86^. These compounds are efficiently integrated into intestinal micelles, absorbed by intestinal epithelial cells, and subsequently assembled into chylomicrons for delivery to the liver. Within hepatic tissue, they are converted into VLDL, which functions as the principal carrier to the developing follicles^87^. Owing to their small size, VLDL particles are able traverse both the basal membrane and granulosa layers of the ovarian follicle, where they bind to specific receptors on the oocyte surface and are internalized through receptor-mediated uptake mechanisms. This pathway enables the efficient deposition of both lipids and carotenoids into the yolk, thereby modulating its lipid profile and pigment concentration^88^. Elevated yolk pigmentation enhances consumer appeal and market value, particularly in markets where deeply colored yolks are preferred^89^. The concurrent decrease in yolk lightness suggests a darkening of yolk intensity, further indicating carotenoid accumulation^24,90^.

Meta-regression analysis revealed that the response of egg quality traits to microalgae supplementation was affected by hen strain, algae inclusion level, and algal species. Variations in strains may represent genetic diversity in lipid transport and reproductive metabolism, influencing yolk deposition and shell formation^91^. The microalgae inclusion level also contributes to enhancing egg quality, likely due to the balanced delivery of nutrients without overwhelming physiological systems. Additionally, microalgae species contribute variably depending on their nutrient profiles—those rich in carotenoids enhanced yolk pigmentation, whereas mineral-dense types boosted shell strength^10,12^.

Dietary microalgae supplementation significantly modified yolk fatty acid profiles by considerably increasing EPA, DHA, and total n-3 PUFA levels while simultaneously lowering n-6 PUFA levels and the n-6/n-3 PUFA ratio. These transitions were observed consistently across studies without significant changes in ALA levels, indicating that yolk enrichment was primarily mediated by the direct incorporation of long-chain n-3 PUFAs from the diet rather than through the endogenous conversion of ALA to EPA/DHA—a pathway acknowledged to be inefficient in birds due to limited Δ6-desaturase activity^92,93^.

Microalgae species such as *S. limacinum*, *N. oculata*, and *I. galbana* are known for their exceptionally high DHA and EPA concentrations, often surpassing the concentrations present in conventional lipid sources like fish oil, flaxseed, or soybean oil^19,58^. In laying hens, fatty acids are integrated into hepatic VLDL during yolk precursor synthesis and then deposited in the oocyte during vitellogenesis^94,95^. VLDL generated in the liver under the action of estrogen are used for egg yolk formation^96^. With the onset of egg production, the VLDL shifts from generic VLDL to VLDLy (yolk targeted)^97^. The high bioavailability of algal DHA and EPA is further boosted by the predominance of these lipids in polar forms—phospholipids and glycolipids—which are more efficiently absorbed through enterocyte transport mechanisms than neutral triglyceride-bound fats^98^.

Concurrent decrease in n-6 PUFA levels and increased yolk n-6/n-3 ratios are particularly noteworthy from a functional food perspective. Eggs rich in n-3 PUFAs and with reduced n-6/n-3 ratios have been associated with anti-inflammatory properties, and their consumption has been linked to decreased risks of cardiovascular, neurodegenerative, and autoimmune disorders in humans^99,100^. Thus, microalgae-enriched eggs serve not only as nutritionally superior foods but also as potential therapeutic dietary components.

Subgroup analyses verified that both microalgae species and laying hen strain significantly influenced the degree of yolk fatty acid enrichment. *Schizochytrium*-based diets caused the highest deposition of DHA and EPA, likely due to their increased n-3 PUFA density and digestibility compared to other microalgal species^18,101^. Strain-wise, Hy-Line and ISA Brown demonstrated greater yolk DHA accumulation than Lohmann Brown, which may be attributed to higher hepatic VLDL receptor expression or apolipoprotein synthesis, promoting more efficient lipid transport to the oocyte^102,103^.

This meta-analysis demonstrated that dietary microalgae supplementation significantly lowered serum concentrations of total cholesterol, AST, and ALT in laying hens, indicating favorable effects on lipid metabolism and liver function^104,105^. Conversely, no significant alterations in serum total protein or triglyceride levels were noted.

The hypocholesterolemic effect of microalgae is in line with a growing body of literature revealing cholesterol-lowering properties in monogastric animals, particularly poultry. Species such as *C. vulgaris*, *A. platensis*, and *S. limacinum* lower circulating cholesterol levels through various mechanisms. One proposed pathway involves the inhibition of 3-hydroxy-3-methyl-glutaryl-coenzyme-A reductase, the rate-limiting enzyme in hepatic cholesterol synthesis^15,106,107^. Furthermore, phytosterols abundant in *Arthrospira* and *Chlorella* structurally resemble cholesterol and compete for micelle formation in the intestine, effectively mitigating dietary cholesterol absorption^85,108^. Additionally, microalgae offer soluble dietary fiber—particularly *β*-glucans and sulfated polysaccharides—which can bind bile acids and elevate their fecal excretion, upregulating hepatic cholesterol catabolism^62,109,110^.

The observed decline in serum AST and ALT levels further support the hepatoprotective role of microalgae. These enzymes are well-established biomarkers of hepatic cellular injury, and their attenuation following microalgae feeding implies preservation of hepatocellular integrity. This effect may be attributed to the high antioxidant content of specific algal species. For instance, *Chlorella* and *Arthrospira* contain high levels of *β*-carotene, tocopherols, and phycocyanin, all of which scavenge ROS and minimize hepatocellular oxidative stress^10,111^. In addition to their antioxidant properties, long-chain n-3 PUFAs such as DHA and EPA—particularly abundant in *Schizochytrium* and *Isochrysis*—regulate hepatic inflammatory responses via downregulation of NF-κB signaling and inhibition of proinflammatory cytokines, including TNF-α and IL-6^19,112^.

In contrast, absence of significant alterations in protein and triglyceride concentrations may indicate complex regulatory dynamics. Protein metabolism in laying hens is closely linked to reproductive phase, dietary amino acid balance, and liver function^113^. The inconsistency in outcomes across studies may be partially explained by variations in the protein digestibility and amino acid composition of different microalgae species. Triglyceride levels, conversely, are impacted by temporal fluctuations in hormone levels and may differ diurnally or in response to stress^114,115^.

This meta-analysis was limited to studies conducted in laying hens under controlled experimental conditions, which may not fully represent practical farm environments. Furthermore, considerable heterogeneity was detected across most evaluated outcomes, which may affect the generalizability of the findings. This heterogeneity likely stems from both biological and methodological factors. Biologically, differences in microalgae species, nutrient composition, and inclusion levels may have influenced the magnitude of responses, as variations in pigment, fatty acid, and antioxidant contents among microalgae are well documented. Methodological variations, including differences in basal diet formulation, experimental duration, housing conditions, and analytical techniques for egg quality and biochemical parameters, may have further contributed to the observed heterogeneity. Despite these differences, subgroup and meta-regression analyses were employed to identify key moderators—such as strain, microalgae level, and species—that influence treatment efficacy. Another strength of this meta-analysis lies in its comprehensive scope, covering not only performance and egg quality, but also yolk fatty acid composition and blood biochemistry, thereby offering a holistic evaluation of dietary microalgae effects. Collectively, this work provides valuable evidence to guide future experimental designs and support functional feed development in layer nutrition. Future research should encompass a broader range of algal species and their bioactive extracts to better elucidate the functional mechanisms underlying their effects on laying hen performance and health.

## Conclusion

This meta-analysis validated that dietary microalgae supplementation can enhance egg production, improve internal and external egg quality traits, fortify yolk with n-3 fatty acids (particularly EPA and DHA), and augment the hepatic function in laying hens. These effects were influenced by strain, microalgae species, and inclusion level, suggesting context-specific responses. Microalgae are a promising functional feed additive for value-added eggs while maintaining egg production performance. These findings are especially relevant for poultry nutritionists, egg producers, and functional food developers who seek to boost animal productivity and meet consumer demand for health-enhanced animal products. Further studies are warranted to optimize inclusion strategies, assess consumer health benefits of enriched eggs under practical production conditions, and encompass a broader range of algal species and their bioactive extracts to better elucidate the functional mechanisms underlying their effects on laying hen performance and health.

## Data Availability

The authors confirm that all data relevant to the findings of this study are presented in the manuscript. Additional datasets generated and analyzed during this research are available from the corresponding author upon justified request.
